# Author Correction: Metabolic heterogeneity of tissue-resident macrophages in homeostasis and during helminth infection

**DOI:** 10.1038/s41467-024-50681-7

**Published:** 2024-07-31

**Authors:** Graham A. Heieis, Thiago A. Patente, Luís Almeida, Frank Vrieling, Tamar Tak, Georgia Perona-Wright, Rick M. Maizels, Rinke Stienstra, Bart Everts

**Affiliations:** 1 Department of Parasitology, Leiden University Medical Center, Albinusdreef 2, 2333 ZA Leiden, The Netherlands; 2 Nutrition, Metabolism and Genomics Group, Division of Human Nutrition and Health, Wageningen University, 6708WE Wageningen, The Netherlands; 3 School of Infection and Immunity, University of Glasgow, 120 University Place, G12 8TA Glasgow, UK

Correction to: *Nature Communications* 10.1038/s41467-023-41353-z, published online 12 September 2023

The original version of this Article contained an error in Fig. 1a, where the table defining the abbreviations of the molecules contained textual mistakes in several lines.

The correct version of Fig. 1 is:
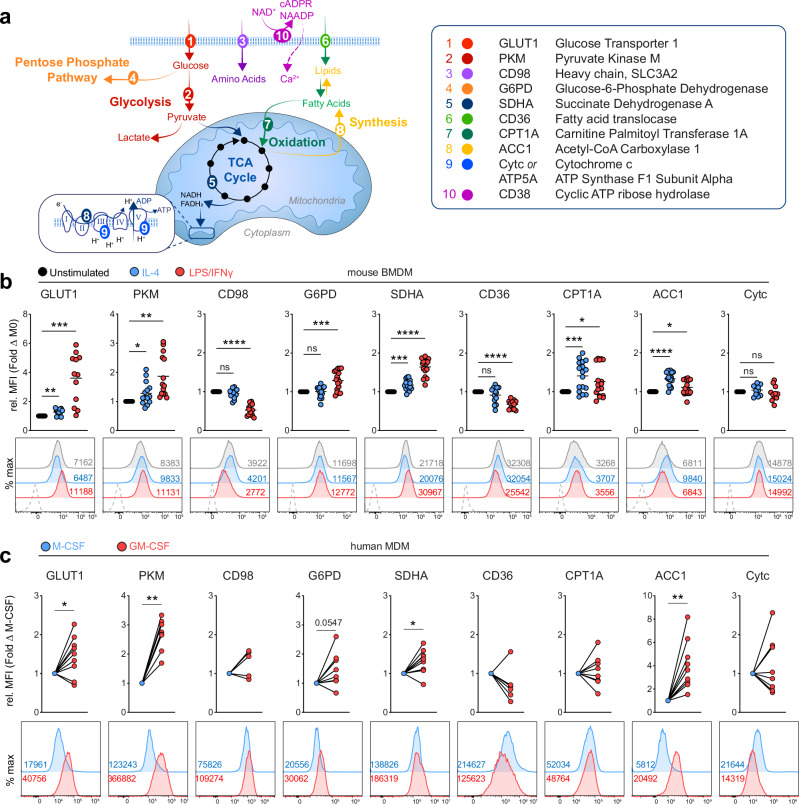


Which replaces the previous incorrect version:
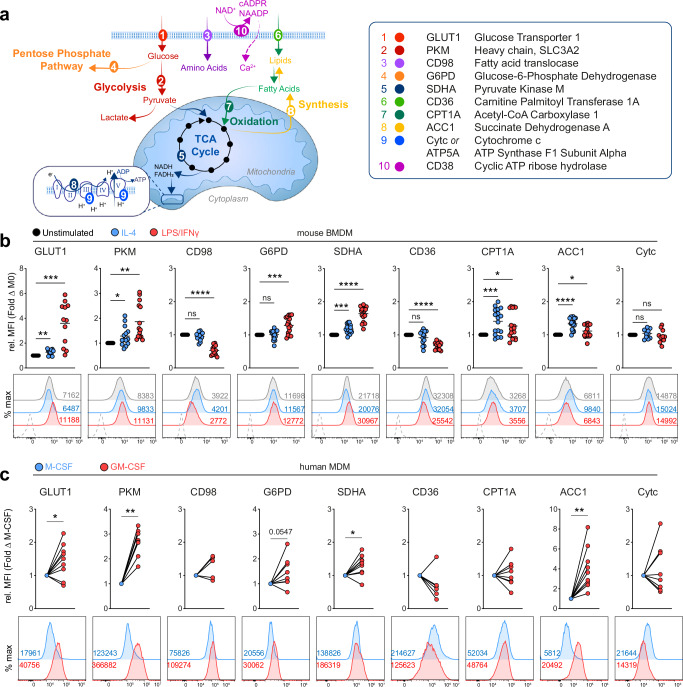


This has been corrected in both the PDF and HTML versions of the Article.

