# Supplementary material for: Metabolic heterogeneity of tissue-resident macrophages in homeostasis and during helminth infection
Source: Nat Commun. 2023 Sep 12;14:5627. doi: 10.1038/s41467-023-41353-z (PMC10497597; doi:10.1038/s41467-023-41353-z)
Supplement: Supplementary file 1 — Supplementary Information [file 41467_2023_41353_MOESM1_ESM.pdf]

Supplementary for:

Metabolic heterogeneity of tissue macrophages in steady-state and helminth infection

Graham A Heieis[1*], Thiago A Patente[1], Luís Almeida[1], Frank Vrieling[2], Tamar Tak[1], Georgia Perona-Wright[3], Rick M. Maizels[3], Rinke Stienstra[2], Bart Everts[1*]

[1]Department of Parasitology, Leiden University Medical Center, Albinusdreef 2, 2333 ZA Leiden, The Netherlands

[2]Nutrition, Metabolism and Genomics Group, Division of Human Nutrition and Health, Wageningen University, 6708WE Wageningen, The Netherlands

[3]School of Infection and Immunity, University of Glasgow, 120 University Place , G12 8TA Glasgow, United Kingdom

**Correspondence: g.a.heieis@lumc.nl, b.everts@lumc.nl*

Supplementary figures: 1-8
Supplementary tables: 1-12
Supplementary methods

Supplementary Figures:

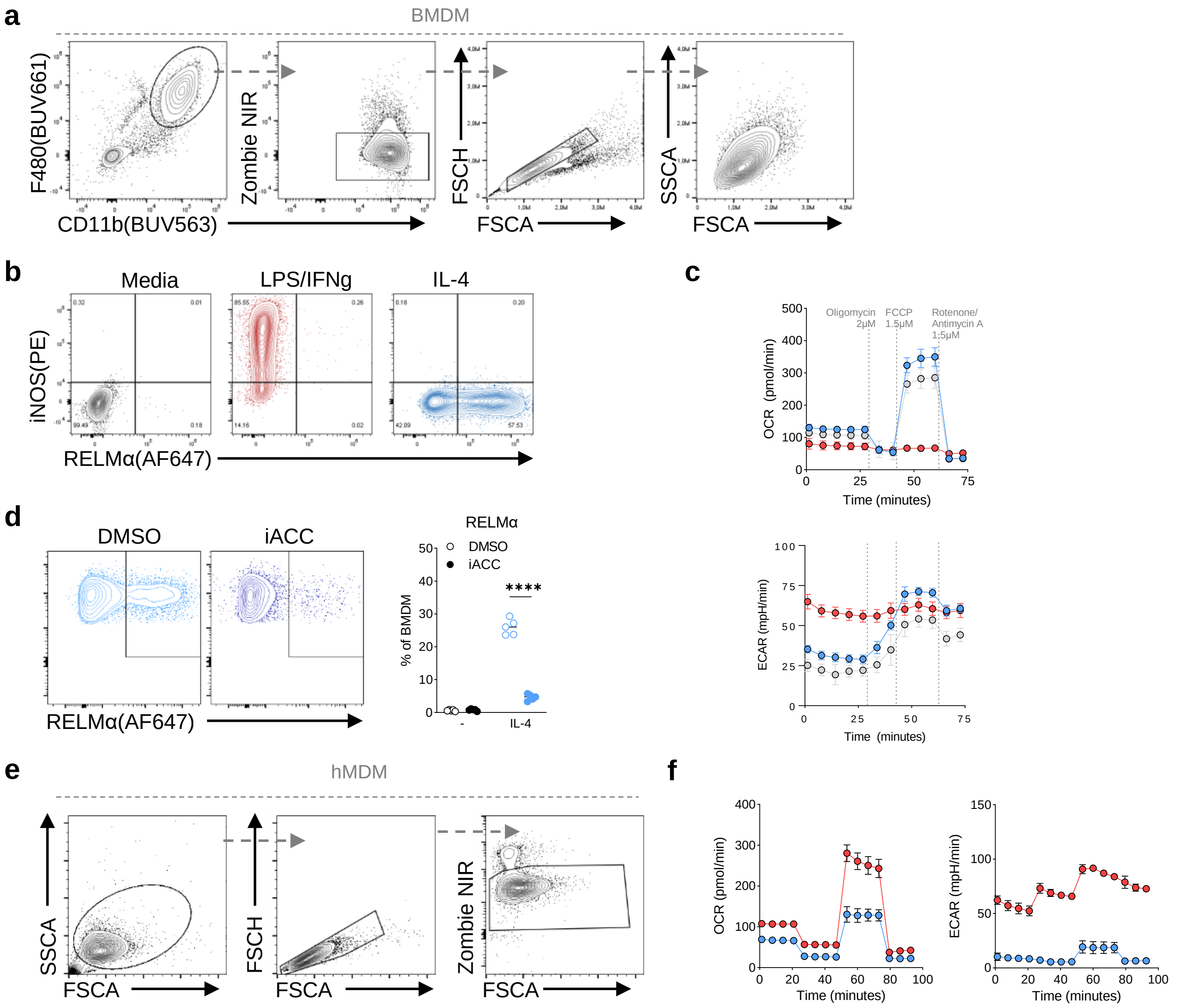

Supplementary Figure 1

(**a**) Representative gating for BMDM after days of culture. (**b**) Polarization of BMDM in response to overnight stimulation with LPS/IFNγ or IL-4. (**c**) Example traces for Seahorse mitochondrial stress test for BMDM, representative of three experiments. (**d**) RELMα expression in control or IL-4 stimulated BMDM cultured with DMSO or ACC inhibitor; data points represent biological replicates from individual mice (n=5), representative of 2 independent experiments. (**e**) Representative scatter-plots for hMDM after 6 days of culture. (**f**) Example Seahorse traces for hMDM, representative of four experiments with 2-3 donors per experiments. Statistics determined by two-tailed unpaired t-test. ****p<0.0001, ***p<0.001, **p<0.01, *p<0.05. Source data are provided as a Source Data file.

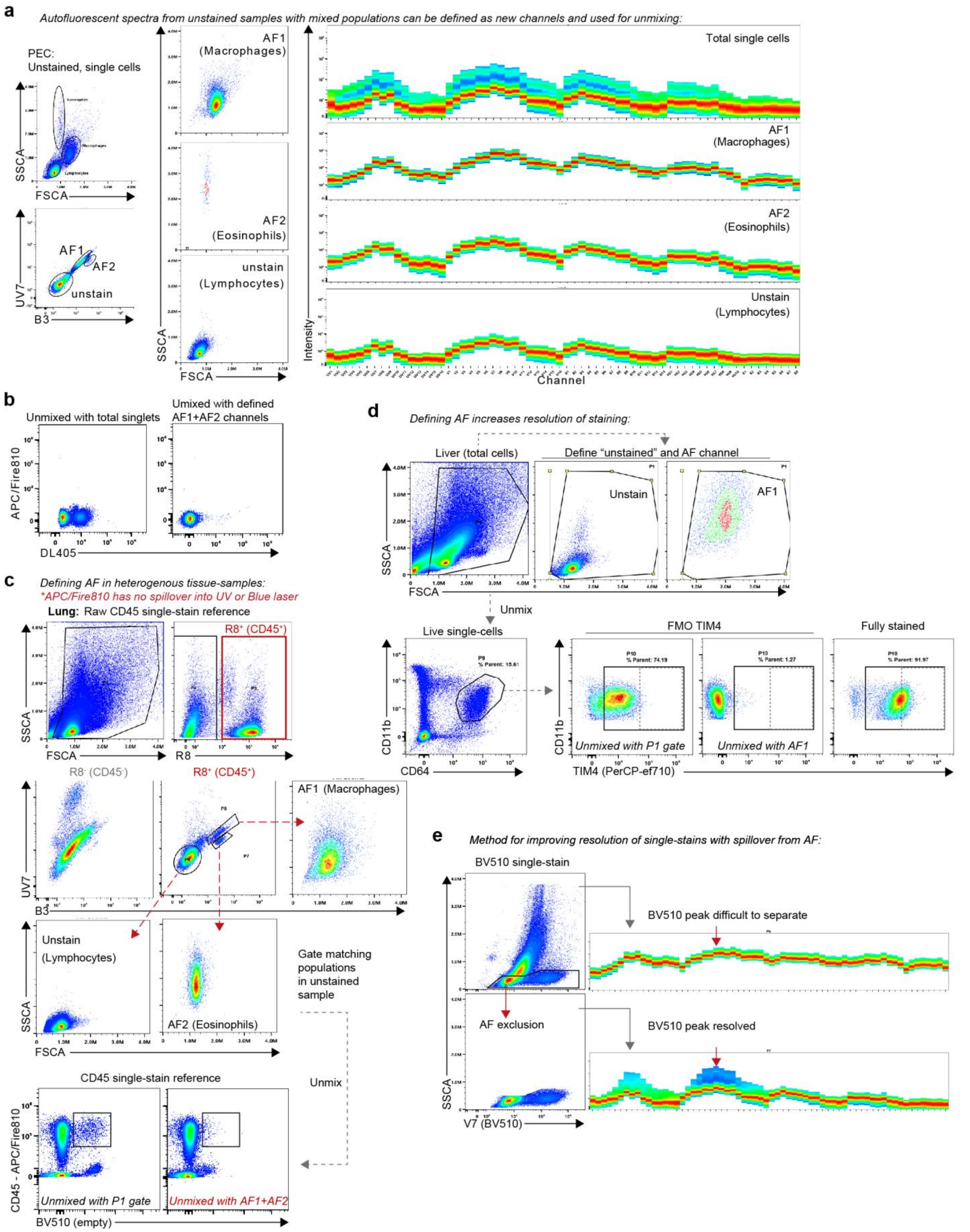

Supplementary Figure 2

(**a**) Distinct scatter profiles and spectral signatures of mphs, lymphocytes and eosinophils in the murine peritoneal cavity. (**b**) Representative unmixing of unstained sample for a channel with high similarity to AF (DL405), and a channel with low similarity to AF (APC-Fire810) unmixing with or without AF channels defined in (a). (**c**) Identification of immune cell AF signatures in stromal tissues using raw worksheet, with the lung as an example, and representative unmixing of unstained samples for BV510 (high AF overlap) with and without AF definition. (**d**) Representative unmixing results for TIM4-PerCP-eFluor710 staining on liver mphs with and without defining extra AF channel. (**e**) Example of increasing resolution of single-stained samples by gating out AF before unmixing.

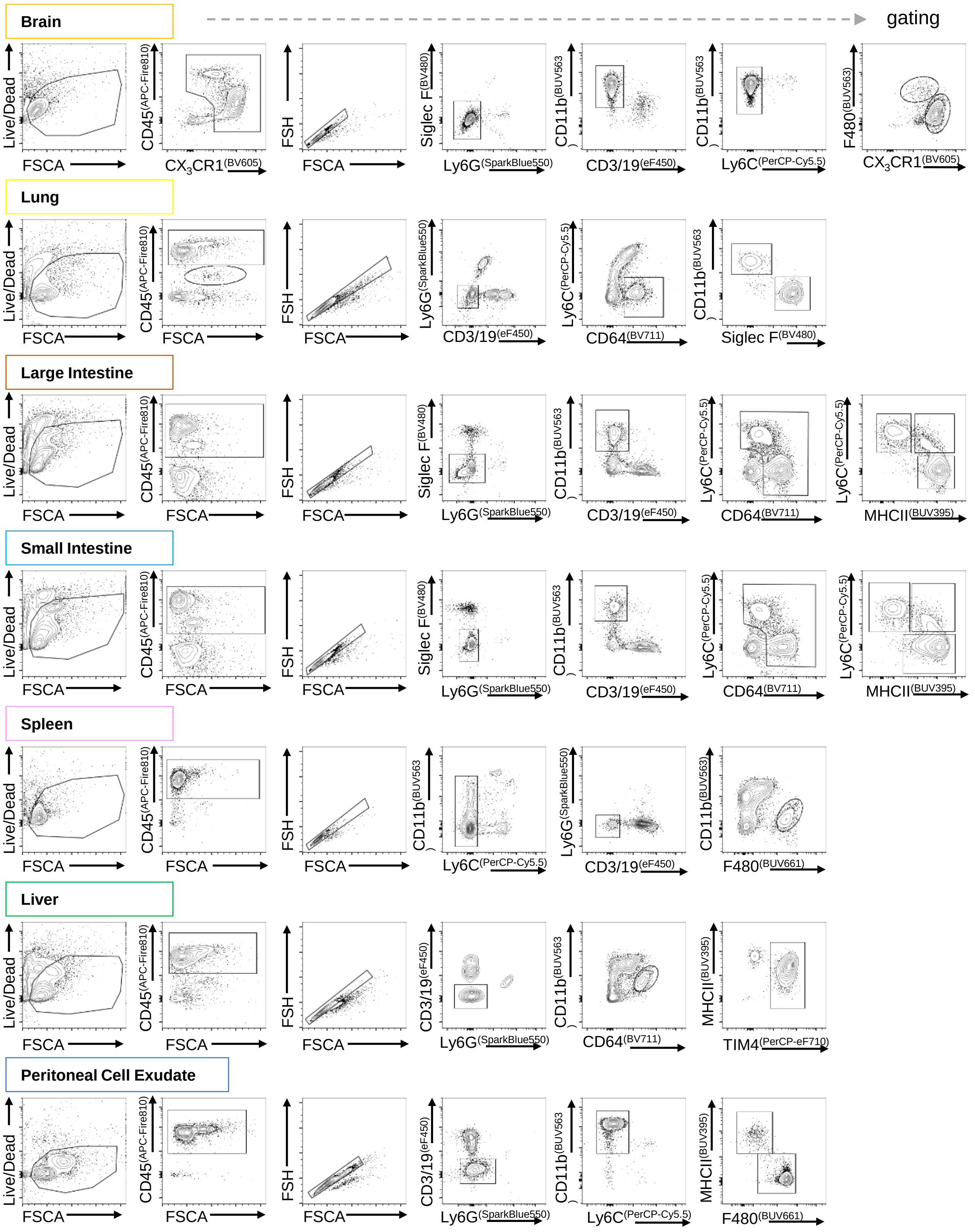

Supplementary Figure 3

Gating strategies for identifying tissue macrophage populations from the whole brain, lung, large and small intestines, spleen, liver and peritoneal cavity.

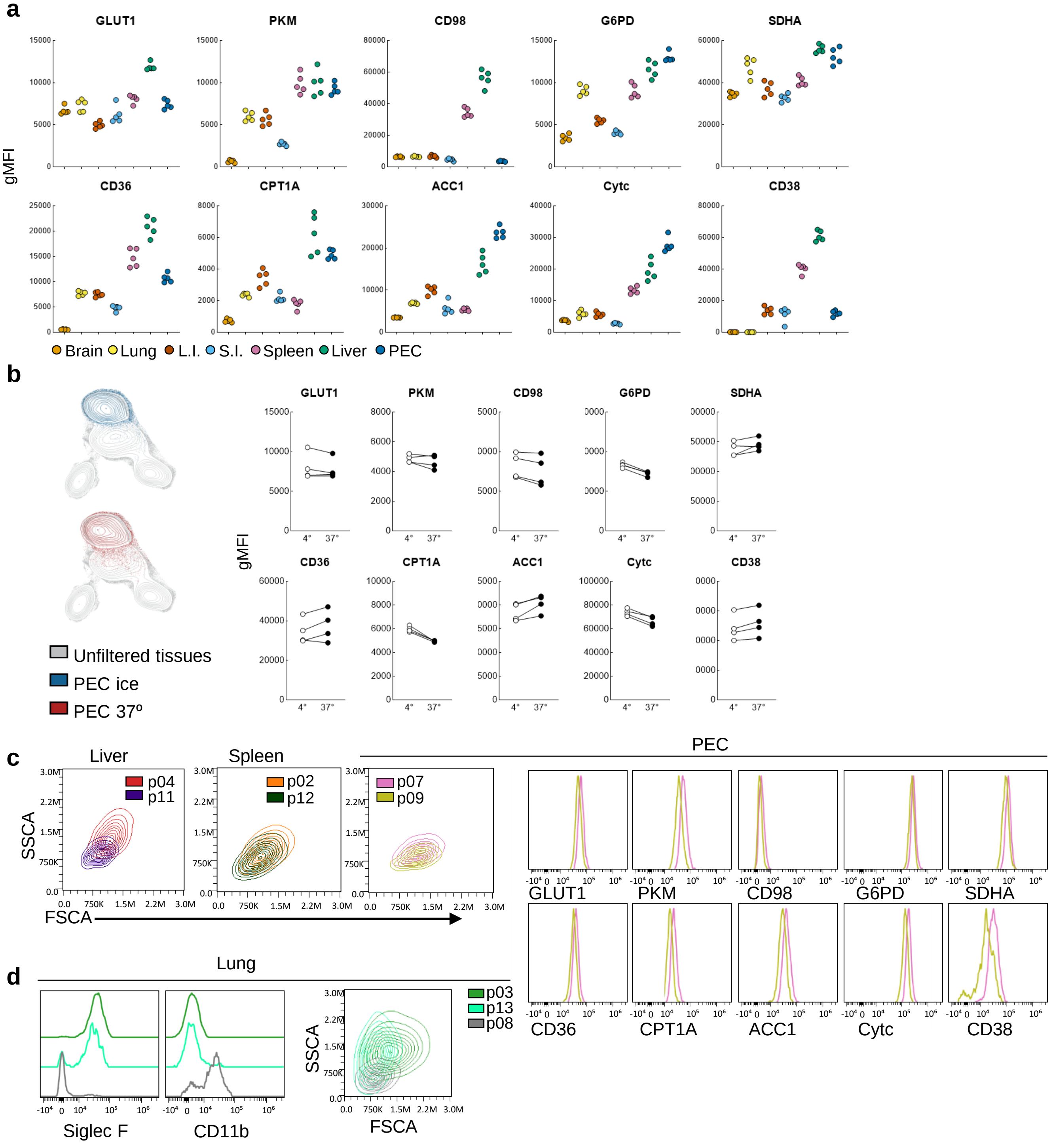

Supplementary Figure 4

(**a**) Comparison of raw gMFIs for metabolic markers across tissue macrophages from 1 of 4 independent experiments. (**b**) PEC samples divided with half stored on ice and the remaining "digested" in RPMI shaking at 37◦C for 30min in parallel to digesting tissues, then assessed for metabolic expression and overlap following UMAP using metabolic targets; data points are individual mice (n=4), representative of 2 experiments. (**c**) Overlay of scatter profiles for prominent clusters in the liver, spleen and PEC and corresponding histograms for metabolic targets in PEC clusters. (**d**) Histograms identifying interstitial and alveolar macrophage clusters and contour plots showing matching scatter profiles. Source data are provided as a Source Data file.

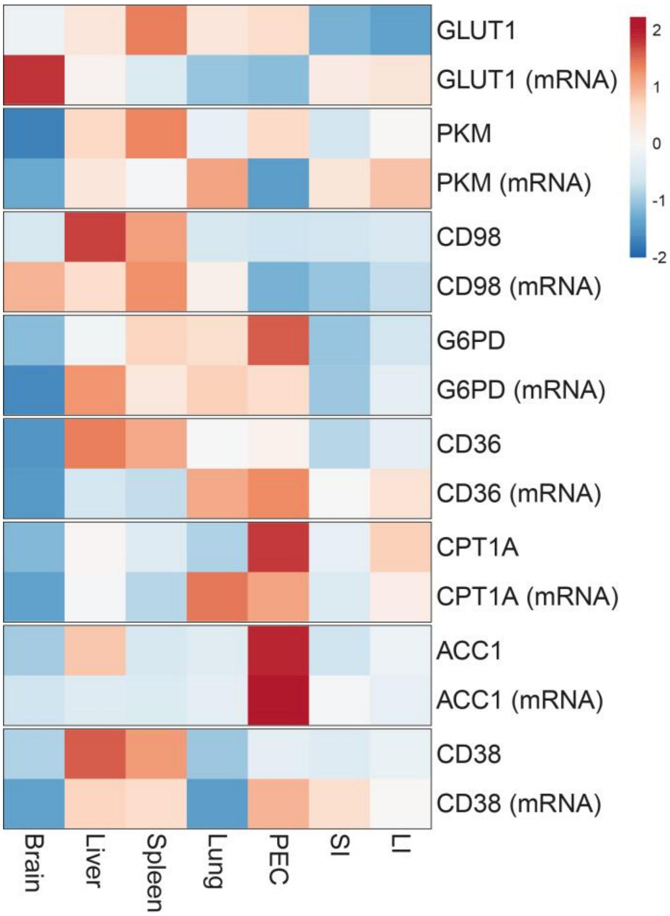

Supplementary Figure 5

Heatmap showing relative expression for gMFI, according to the mean, of metabolic targets detected by spectral flow cytometry, compared to mean expression (log base 2) from bulk RNA-seq data generated by Lavin *et al*[24] , generated using ClustVis[57].

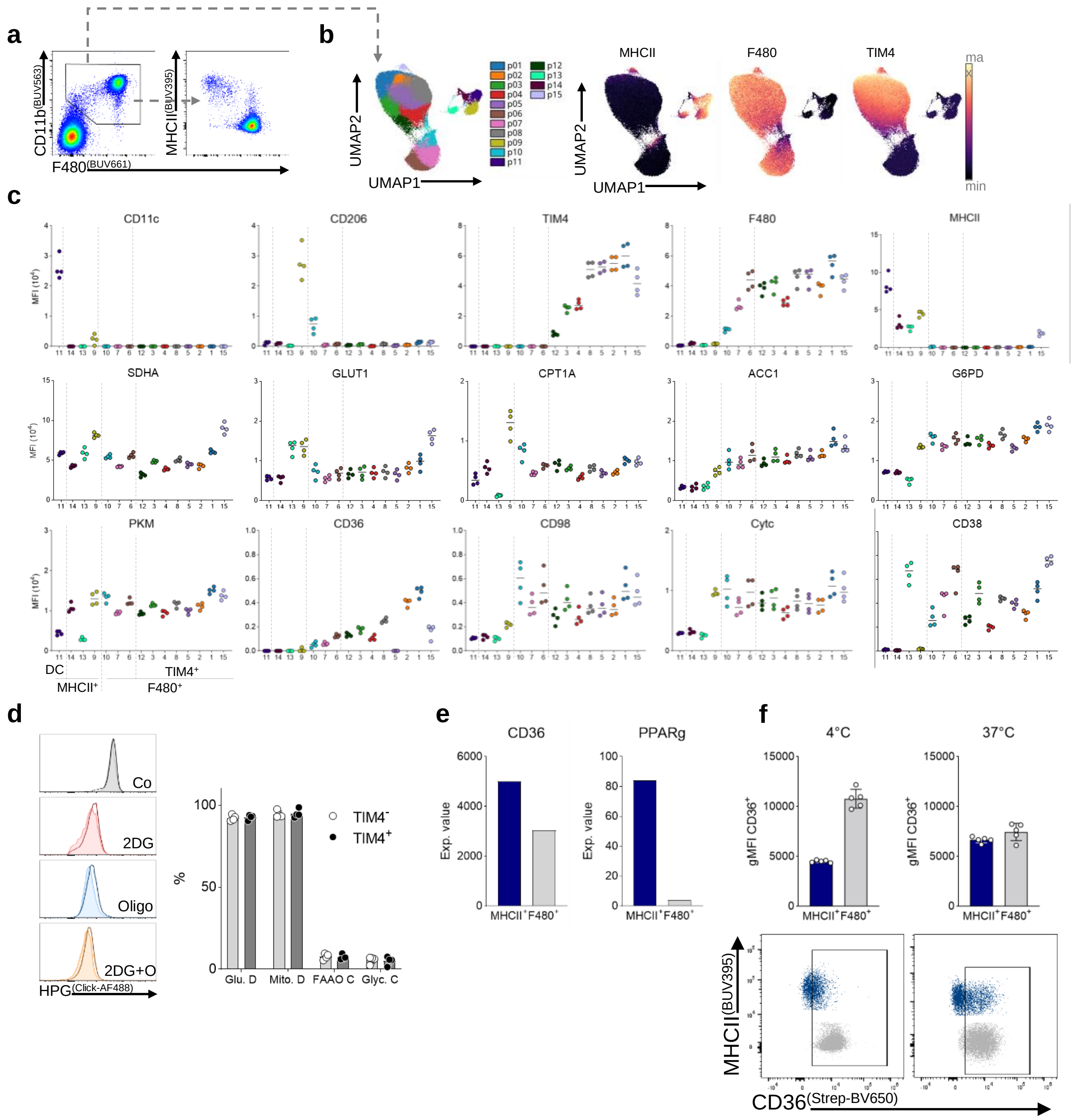

Supplementary Figure 6

(**a**) Gating for UMAP and phenograph analysis of PEC macrophages, as shown in (**b**) generated using immune (CD11b, F480, MHCII, CD11c, TIM4, CD206, CX_3CR1, Ly6C, CD301) and metabolic markers. (**c**) Expression of immune and metabolic markers for identified peritoneal macrophage clusters; corresponding data to Figure 3c. (**d**) Overlaid histograms and graphed data comparing HPG uptake of $TIM4^-$ and $TIM4^+$ peritoneal macrophages, corresponding data to Figure 3d. (**e**) Normalized gene expression of CD36 and PPARγ in $MHCII^{Hi}$ and $F480^{Hi}$ macrophages, taken from ImmGen Gene Skyline data browser. (**f**) Comparison of CD36 expression between $MHCII^{Hi}$ and $F480^{Hi}$ macrophages, gated on $CD36^+$ cells, when stained after 20 minutes of incubation at 37◦C or kept on ice, data points are individual mice (n=5, mean±SD). Source data are provided as a Source Data file.

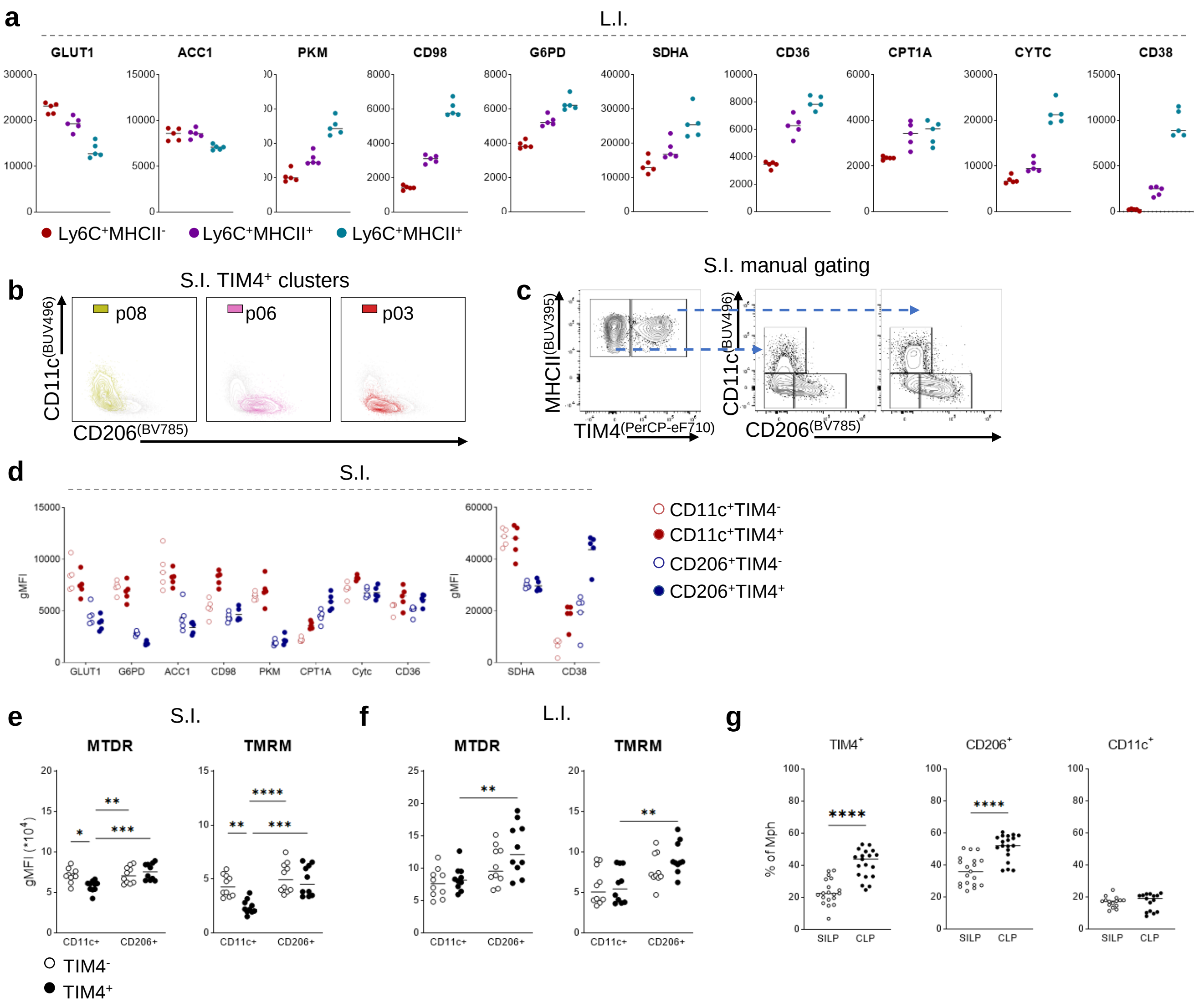

Supplementary Fig. 7

(**a**) Metabolic target expression in colonic monocytes, intermediate monocytes/macrophages, and mature macrophages, representative of 4 experiments with n=5 mice. (**b**) Contour plots of CD11c and CD206 expression of TIM4 phenograph clusters identified from the small intestine. (**c**) Manual gating of CD11c$^+$ or CD206$^+$ cells within TIM4+ small intestinal macrophages. (**d**) Comparison of metabolic protein expression (gMFI) between CD11c$^+$ and CD206$^+$ cells within TIM4$^-$ and TIM4$^+$ macrophages, representative of 4 experiments with n=5 mice. (**e**) MitoTracker DeepRed and TMRM staining of macrophages from the small and (**f**) large intestine, according to TIIM4, CD11c or CD206 expression as gated in (**c**), pooled from 2 independent experiments (n=5 mice per experiment). (**g**) Frequency of TIM4$^+$, CD206$^+$ and CD11c$^+$ macrophages in the small and large intestine pooled from 3 independent experiments (n=4-5 mice per experiment). Statistics calculated using two-way ANOVA with Tukey test for multiple comparisons (e,f) or two-tailed unpaired t-test (g). ****p<0.0001, ***p<0.001, **p<0.01, *p<0.05. Source data are provided as a Source Data file.

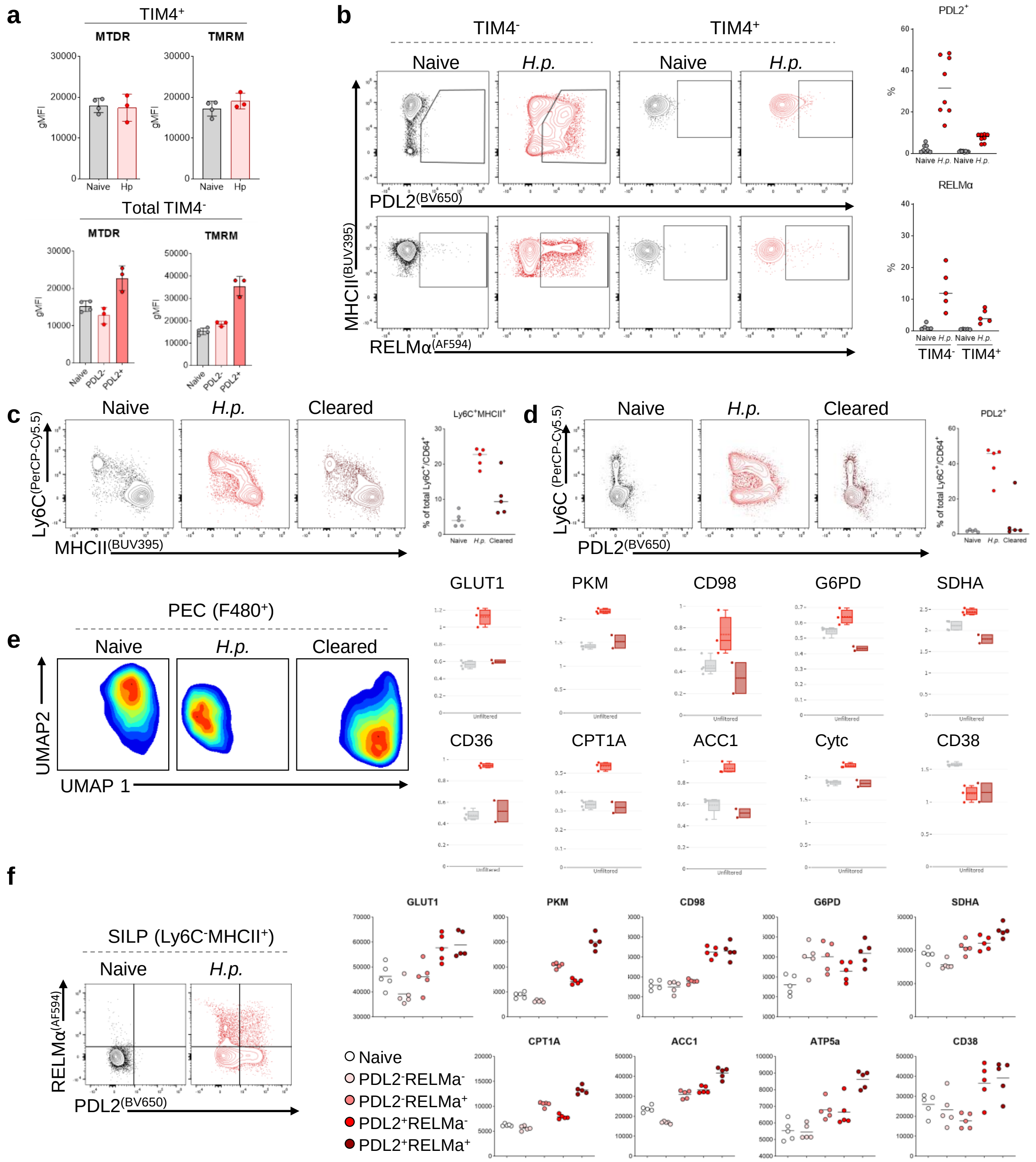

Supplementary Figure 8

(**a**) Quantified MitoTracker DeepRed and TMRM staining on small intestine macrophages from naïve (n=4) or Hp infected (n=3) mice, one experiment represented (mean±SD). (**b**) Representative staining and frequencies for alternative activation markers in TIM4⁻ monocytes/macrophages and TIM4⁺ macrophages from the small intestine following *H. polygyrus* infection, representative of, or pooled from, 2 experiments with 3-5 mice per group. (**c**) Flow plots displaying monocyte waterfall in naïve, infected and cleared mice, and corresponding frequencies of monocytes, intermediate and mature macrophages. (**d**) Corresponding flow plots for Ly6C and PDL2 expression and frequency of total PDL2⁺ cells, individual mice shown (n=5 per group), representative of 2 experiments. (**e**) UMAP and relative metabolic expression in peritoneal macrophages before, during or after infection, individual mice shown and representative of 2 experiments (n=2-5/group, 25-75th percentile with median, min and max shown). (**f**) Gating used to identify PDL2⁺/RELMα⁺ macrophages, used for Fig. 6h, and gMFI for metabolic targets in each quadrant, individual mice shown (n=5 per group), representative of 2 experiments. Source data are provided as a Source Data file.

Supplementary Tables

Supplementary Table 1: Flow cytometry reagents and staining order

Target	Fluor	Company	Cat. #	Dilution
Live/dead stain				
Fixable viability kit	Zombie NIR	BioLegend	423106	1000x
Surface pre-fix				
CD11c	BUV496	BD Bioscience	750450	400x
CD206	BV785	BioLegend	141729	400x
CX_3CR1	BV605	BioLegend	149027	100x
CD36	AF700	BioLegend	56-0362-80	100x
	Biotin	BioLegend	102604	100x
CD19 *or*	ef450	ThermoFisher	48-0193-82	100x
B220	BV510	BioLegend	103248	100x
Surface post-fix				
CD45	APC/Fire810	BioLegend	103173	2000x
CD11b	BUV563	BD Biosciences	741242	10000x
F4/80	BUV661	BD Biosciences	750643	*200x
CD64	BV711	BioLegend	139311	100x
TIM4	PerCP-ef710	ThermoFisher	46-5866-82	**10000x
MHCII	BUV395	BD Biosciences	743876	3000x
PDL1	BUV737	BD Biosciences	741877	100x
PDL2	BV650	BD Biosciences	740624	100x
Ly6G	SB550	BioLegend	108467	1000x
Siglec F	BV480	BD Biosciences	746668	400x
Ly6C	PerCP-Cy5.5	BioLegend	128012	1000x
CD3	BV750	BioLegend	100249	200x
	eF450	ThermoFisher	48-0032-82	100x
CD98	BUV615	BD Biosciences	752360	400x
CD38	BUV805	BD Biosciences	741955	400x
Streptavidin	BV570 or	BioLegend	405227	400x
	BV650	BioLegend	405232	400x
Intracellular stain				
iNOS	PE	BioLegend	696806	10000x
RELM-alpha primary	-	PeproTech	500-P214	0.5µg/ml
Goat αRb secondary	AF594	ThermoFisher		1000x
	AF647	ThermoFisher		

Metabolic Targets				
Glut1	BSA/Azide free	Abcam	ab252403	1000x
(Lightning link)	DL405		ab201798	
PKM	BSA/Azide free	Abcam	ab206129	400x
(Lightning link)	PE		ab102918	
SDHA	BSA/Azide free	Abcam	ab240098	2000x
(Lightning link)	AF647		ab269823	
CPT1A	BSA/Azide free	Abcam	ab235841	200x
(Lightning link)	PE/Cy5		ab102893	
ACC1	BSA/Azide free	Abcam	ab272704	
(Lightning link)	AF488		ab236553	200x
(Lightning link)	PE/Cy7		ab102903	1000x
Cytc	BSA/Azide free	Abcam	ab237966	1000x
(Lightning link)	PE/Cy7		ab102903	
ATP5a	BSA/Azide free	Abcam	ab231692	200x
(Lightning link)	AF488		ab236553	
G6PD	BSA/Azide free	Abcam	ab231828	1000x
(Lightning link)	APC/Cy7		ab102859	

*F4/80 = 6000x for PEC

**TIM4 = 200x for intestines, 1000x for liver

Supplementary Table 2: Statistical comparisons for tissue ACC1 expression

Dunn's multiple comparisons test	Significant?	Summary	Adjusted P Value
Brain vs. Lung	No	ns	0,0622
Brain vs. cLP	Yes	****	<0,0001
Brain vs. siLP	No	ns	0,4586
Brain vs. Spleen	No	ns	0,0608
Brain vs. Liver	Yes	****	<0,0001
Brain vs. PEC	Yes	****	<0,0001
Lung vs. cLP	No	ns	0,1459
Lung vs. siLP	No	ns	>0,9999
Lung vs. Spleen	No	ns	>0,9999
Lung vs. Liver	Yes	****	<0,0001
Lung vs. PEC	Yes	****	<0,0001
cLP vs. siLP	Yes	*	0,0121
cLP vs. Spleen	No	ns	0,5574
cLP vs. Liver	No	ns	0,8864
cLP vs. PEC	Yes	*	0,0121
siLP vs. Spleen	No	ns	>0,9999
siLP vs. Liver	Yes	****	<0,0001
siLP vs. PEC	Yes	****	<0,0001
Spleen vs. Liver	Yes	***	0,0010
Spleen vs. PEC	Yes	****	<0,0001
Liver vs. PEC	No	ns	>0,9999

Supplementary Table 3: Statistical comparisons for tissue SDHA expression

Dunn's multiple comparisons test	Significant?	Summary	Adjusted P Value
Brain vs. Lung	No	ns	>0,9999
Brain vs. cLP	No	ns	>0,9999
Brain vs. siLP	No	ns	>0,9999
Brain vs. Spleen	Yes	*	0,0202
Brain vs. Liver	No	ns	>0,9999
Brain vs. PEC	No	ns	>0,9999
Lung vs. cLP	No	ns	>0,9999
Lung vs. siLP	No	ns	>0,9999
Lung vs. Spleen	Yes	****	<0,0001
Lung vs. Liver	Yes	**	0,0021
Lung vs. PEC	Yes	**	0,0011
cLP vs. siLP	No	ns	0,8614
cLP vs. Spleen	Yes	**	0,0052
cLP vs. Liver	No	ns	0,7902
cLP vs. PEC	No	ns	0,5420
siLP vs. Spleen	Yes	****	<0,0001
siLP vs. Liver	Yes	***	0,0008
siLP vs. PEC	Yes	***	0,0004
Spleen vs. Liver	No	ns	>0,9999
Spleen vs. PEC	No	ns	>0,9999
Liver vs. PEC	No	ns	>0,9999

Supplementary Table 4: Statistical comparisons for tissue G6PD expression

Dunn's multiple comparisons test	Significant?	Summary	Adjusted P Value
Brain vs. Lung	Yes	****	<0,0001
Brain vs. cLP	Yes	*	0,0382
Brain vs. siLP	No	ns	>0,9999
Brain vs. Spleen	Yes	****	<0,0001
Brain vs. Liver	Yes	****	<0,0001
Brain vs. PEC	Yes	****	<0,0001
Lung vs. cLP	No	ns	0,7750
Lung vs. siLP	Yes	***	0,0005
Lung vs. Spleen	No	ns	>0,9999
Lung vs. Liver	No	ns	>0,9999
Lung vs. PEC	Yes	**	0,0028
cLP vs. siLP	No	ns	0,6561
cLP vs. Spleen	No	ns	0,1819
cLP vs. Liver	No	ns	0,2118
cLP vs. PEC	Yes	****	<0,0001
siLP vs. Spleen	Yes	****	<0,0001
siLP vs. Liver	Yes	****	<0,0001
siLP vs. PEC	Yes	****	<0,0001
Spleen vs. Liver	No	ns	>0,9999
Spleen vs. PEC	No	ns	0,1182
Liver vs. PEC	Yes	*	0,0179

Supplementary Table 5: Statistical comparisons for tissue CD98 expression

Dunn's multiple comparisons test	Significant?	Summary	Adjusted P Value
Brain vs. Lung	No	ns	>0,9999
Brain vs. cLP	No	ns	>0,9999
Brain vs. siLP	No	ns	0,0966
Brain vs. Spleen	No	ns	0,0897
Brain vs. Liver	Yes	***	0,0004
Brain vs. PEC	No	ns	0,2815
Lung vs. cLP	No	ns	>0,9999
Lung vs. siLP	No	ns	0,0613
Lung vs. Spleen	Yes	*	0,0286
Lung vs. Liver	Yes	****	<0,0001
Lung vs. PEC	No	ns	0,2070
cLP vs. siLP	No	ns	0,5256
cLP vs. Spleen	Yes	**	0,0022
cLP vs. Liver	Yes	****	<0,0001
cLP vs. PEC	No	ns	>0,9999
siLP vs. Spleen	Yes	****	<0,0001
siLP vs. Liver	Yes	****	<0,0001
siLP vs. PEC	No	ns	>0,9999
Spleen vs. Liver	No	ns	>0,9999
Spleen vs. PEC	Yes	****	<0,0001
Liver vs. PEC	Yes	****	<0,0001

Supplementary Table 6: Statistical comparisons for tissue CD36 expression

Dunn's multiple comparisons test	Significant?	Summary	Adjusted P Value
Brain vs. Lung	Yes	**	0,0091
Brain vs. cLP	No	ns	0,4853
Brain vs. siLP	No	ns	>0,9999
Brain vs. Spleen	Yes	****	<0,0001
Brain vs. Liver	Yes	****	<0,0001
Brain vs. PEC	Yes	***	0,0009
Lung vs. cLP	No	ns	>0,9999
Lung vs. siLP	No	ns	0,1006
Lung vs. Spleen	No	ns	0,0953
Lung vs. Liver	No	ns	0,4410
Lung vs. PEC	No	ns	>0,9999
cLP vs. siLP	No	ns	>0,9999
cLP vs. Spleen	Yes	***	0,0009
cLP vs. Liver	Yes	**	0,0040
cLP vs. PEC	No	ns	0,7724
siLP vs. Spleen	Yes	****	<0,0001
siLP vs. Liver	Yes	****	<0,0001
siLP vs. PEC	Yes	*	0,0103
Spleen vs. Liver	No	ns	>0,9999
Spleen vs. PEC	No	ns	0,5074
Liver vs. PEC	No	ns	>0,9999

Supplementary Table 7: Statistical comparisons for tissue GLUT1 expression

Dunn's multiple comparisons test	Significant?	Summary	Adjusted P Value
Brain vs. Lung	Yes	**	0,0027
Brain vs. cLP	Yes	**	0,0033
Brain vs. siLP	Yes	**	0,0014
Brain vs. Spleen	No	ns	>0,9999
Brain vs. Liver	No	ns	>0,9999
Brain vs. PEC	No	ns	>0,9999
Lung vs. cLP	No	ns	>0,9999
Lung vs. siLP	No	ns	>0,9999
Lung vs. Spleen	Yes	****	<0,0001
Lung vs. Liver	Yes	****	<0,0001
Lung vs. PEC	Yes	****	<0,0001
cLP vs. siLP	No	ns	>0,9999
cLP vs. Spleen	Yes	****	<0,0001
cLP vs. Liver	Yes	****	<0,0001
cLP vs. PEC	Yes	****	<0,0001
siLP vs. Spleen	Yes	****	<0,0001
siLP vs. Liver	Yes	****	<0,0001
siLP vs. PEC	Yes	****	<0,0001
Spleen vs. Liver	No	ns	>0,9999
Spleen vs. PEC	No	ns	>0,9999
Liver vs. PEC	No	ns	>0,9999

Supplementary Table 8: Statistical comparisons for tissue PKM expression

Dunn's multiple comparisons test	Significant?	Summary	Adjusted P Value
Brain vs. Lung	Yes	**	0,0036
Brain vs. cLP	Yes	**	0,0026
Brain vs. siLP	No	ns	>0,9999
Brain vs. Spleen	Yes	****	<0,0001
Brain vs. Liver	Yes	****	<0,0001
Brain vs. PEC	Yes	****	<0,0001
Lung vs. cLP	No	ns	>0,9999
Lung vs. siLP	No	ns	0,7099
Lung vs. Spleen	Yes	*	0,0408
Lung vs. Liver	Yes	**	0,0042
Lung vs. PEC	Yes	***	0,0002
cLP vs. siLP	No	ns	0,5702
cLP vs. Spleen	No	ns	0,0532
cLP vs. Liver	Yes	**	0,0059
cLP vs. PEC	Yes	***	0,0003
siLP vs. Spleen	Yes	****	<0,0001
siLP vs. Liver	Yes	****	<0,0001
siLP vs. PEC	Yes	****	<0,0001
Spleen vs. Liver	No	ns	>0,9999
Spleen vs. PEC	No	ns	>0,9999
Liver vs. PEC	No	ns	>0,9999

Supplementary Table 9: Statistical comparisons for tissue CPT1A expression

Dunn's multiple comparisons test	Significant?	Summary	Adjusted P Value
Brain vs. cLP	Yes	***	0,0002
Brain vs. siLP	No	ns	0,1012
Brain vs. Spleen	No	ns	>0,9999
Brain vs. Liver	Yes	****	<0,0001
Brain vs. PEC	Yes	****	<0,0001
Lung vs. cLP	No	ns	>0,9999
Lung vs. siLP	No	ns	>0,9999
Lung vs. Spleen	No	ns	>0,9999
Lung vs. Liver	No	ns	>0,9999
Lung vs. PEC	Yes	***	0,0004
cLP vs. siLP	No	ns	>0,9999
cLP vs. Spleen	No	ns	0,1552
cLP vs. Liver	No	ns	>0,9999
cLP vs. PEC	Yes	*	0,0158
siLP vs. Spleen	No	ns	>0,9999
siLP vs. Liver	No	ns	0,5777
siLP vs. PEC	Yes	****	<0,0001
Spleen vs. Liver	No	ns	0,0528
Spleen vs. PEC	Yes	****	<0,0001
Liver vs. PEC	No	ns	0,0606

Supplementary Table 10: Statistical comparisons for tissue Cytc expression

Dunn's multiple comparisons test	Significant?	Summary	Adjusted P Value
Brain vs. Lung	No	ns	0,9698
Brain vs. cLP	Yes	**	0,0018
Brain vs. siLP	No	ns	0,8670
Brain vs. Spleen	Yes	****	<0,0001
Brain vs. Liver	Yes	****	<0,0001
Brain vs. PEC	Yes	****	<0,0001
Lung vs. cLP	No	ns	0,7169
Lung vs. siLP	No	ns	>0,9999
Lung vs. Spleen	Yes	**	0,0059
Lung vs. Liver	Yes	****	<0,0001
Lung vs. PEC	Yes	****	<0,0001
cLP vs. siLP	No	ns	0,8133
cLP vs. Spleen	No	ns	>0,9999
cLP vs. Liver	No	ns	0,0781
cLP vs. PEC	Yes	****	<0,0001
siLP vs. Spleen	Yes	**	0,0070
siLP vs. Liver	Yes	****	<0,0001
siLP vs. PEC	Yes	****	<0,0001
Spleen vs. Liver	No	ns	>0,9999
Spleen vs. PEC	No	ns	0,2121
Liver vs. PEC	No	ns	>0,9999

Supplementary Table 11: Statistical comparisons for tissue CD38 expression

Dunn's multiple comparisons test	Significant?	Summary	Adjusted P Value
MG vs. AlvM	No	ns	>0,9999
MG vs. CLP	No	ns	0,1593
MG vs. SILP	Yes	*	0,0204
MG vs. RPM	Yes	****	<0,0001
MG vs. KC	Yes	****	<0,0001
MG vs. LPM	No	ns	0,0801
AlvM vs. CLP	Yes	*	0,0238
AlvM vs. SILP	Yes	**	0,0014
AlvM vs. RPM	Yes	****	<0,0001
AlvM vs. KC	Yes	****	<0,0001
AlvM vs. LPM	Yes	**	0,0093
CLP vs. SILP	No	ns	>0,9999
CLP vs. RPM	No	ns	0,0504
CLP vs. KC	Yes	**	0,0014
CLP vs. LPM	No	ns	>0,9999
SILP vs. RPM	No	ns	0,3372
SILP vs. KC	Yes	*	0,0227
SILP vs. LPM	No	ns	>0,9999
RPM vs. KC	No	ns	>0,9999
RPM vs. LPM	No	ns	0,1031
KC vs. LPM	Yes	**	0,0039

Supplementary Table 12: Correlation coefficients for PDL1 and metabolic marker expression using simple linear regression

	GLUT1	PKM	CD98	G6PD	SDHA	CD36	CPT1A	Cytc	CD38
Exp. 1	0.71	0.13	0.28	0.54	0.45	0.23	0.06	0.12	0.56
Exp. 2	0.59	0.58	0.10	0.82	0.83	0.61	0.59	0.70	0.71
Exp. 3	0.001	0.76	0.46	0.28	0.22	0.63	0.60	0.66	0.006
Exp. 4	0.40	0.67	0.005	0.80	0.31	0.95	N/A	0.77	0.91

Supplementary Methods:
Unmixing with SpectroFlow

Reference controls

Reference controls were stained simultaneously in parallel to the corresponding staining step for the given marker. Beads were used, as we found that this reduced the complexity resulting from AF, and improved resolution of macrophage markers. Cells were used only if beads were not suitable for the antibody isotype, or if the brightness on cells was consistently brighter than on the beads. Cells were used to generate references for all intracellular metabolic targets. Single stain references were re-used for subsequent experiments up to ~2 months, or after servicing of the cytometer.

Defining AF/unstain and unmixing

The following is a guideline for defining AF, however exact protocol is subject to each experiment/tissue:

- Create a gate to exclude debris, and second gate to exclude aggregates (single-cell gate)
- Within single cell gate, exclude UV7-high cells (P3 gate)
    - This removes large proportion of dead/non-immune cells, as seen in Supplementary Fig. 2c
- Within P3, use 2-D plot with aid of spectrogram to cycle through common peak AF channels to identify if multiple populations are present
    - Often, in our hands a combination of B3, UV7 or V7 will identify macrophages and eosinophils
    - Confirm homogeneity of population with spectrogram
- Once AF population has been identified, right click gated area and export as FCS
- Similarly for unstained, use 2-D plot of P3 but gate most AF low population
- Create a new AF channel (Library -> Fluorescent tags -> select laser group -> "Add" -> define and label AF channel) and add tag to experiment setup
    - Add corresponding reference under "edit"->"groups"
- Import exported FCS files to new AF channel and unstain reference
- "Unmix"
    - In general, macrophage AF has similar overall spectra as lymphocytes, so it is not necessary to additionally select the "Autofluorescence as tag" toggle

In the event of multiple AF populations it is possible to check their resulting complexity:

- Aim to gate the most negative population possible within total cells (debris gate – spectrum should be as "flat" as possible) and export as FCS
- Set the "debris" gate as the unstained and import AF populations into reference group
- "Unmix", define references and select "QC Controls" tag and "similarity matrix"
    - Click view similarity index to view the complexity between defined AF channels – anything less than 0.98 can be used as a separate tag

Note: defining multiple channels will not always improve unmixing, so a certain trial and error is required to determine optimal AF channels and unmixing.
