# Supplementary material for: Metabolic heterogeneity of tissue-resident macrophages in homeostasis and during helminth infection
Source: Nat Commun. 2023 Sep 12;14:5627. doi: 10.1038/s41467-023-41353-z (PMC10497597; doi:10.1038/s41467-023-41353-z)
Supplement: Supplementary file 3 — Reporting Summary [file 41467_2023_41353_MOESM3_ESM.pdf]

nature portfolio

Corresponding author(s): Bart Everts
Graham Heieis

Last updated by author(s): Aug 10, 2023

Reporting Summary

Nature Portfolio wishes to improve the reproducibility of the work that we publish. This form provides structure for consistency and transparency in reporting. For further information on Nature Portfolio policies, see our Editorial Policies and the Editorial Policy Checklist.

Statistics

For all statistical analyses, confirm that the following items are present in the figure legend, table legend, main text, or Methods section.

n/a	Confirmed	
☐	☒	The exact sample size (*n*) for each experimental group/condition, given as a discrete number and unit of measurement
☐	☒	A statement on whether measurements were taken from distinct samples or whether the same sample was measured repeatedly
☐	☒	The statistical test(s) used AND whether they are one- or two-sided<br>*Only common tests should be described solely by name; describe more complex techniques in the Methods section.*
☒	☐	A description of all covariates tested
☐	☒	A description of any assumptions or corrections, such as tests of normality and adjustment for multiple comparisons
☐	☒	A full description of the statistical parameters including central tendency (e.g. means) or other basic estimates (e.g. regression coefficient) AND variation (e.g. standard deviation) or associated estimates of uncertainty (e.g. confidence intervals)
☐	☒	For null hypothesis testing, the test statistic (e.g. *F*, *t*, *r*) with confidence intervals, effect sizes, degrees of freedom and *P* value noted<br>*Give P values as exact values whenever suitable.*
☒	☐	For Bayesian analysis, information on the choice of priors and Markov chain Monte Carlo settings
☒	☐	For hierarchical and complex designs, identification of the appropriate level for tests and full reporting of outcomes
☒	☐	Estimates of effect sizes (e.g. Cohen's *d*, Pearson's *r*), indicating how they were calculated

Our web collection on statistics for biologists contains articles on many of the points above.

Software and code

Policy information about availability of computer code

Data collection	Cytek SpectroFlo version 3 was used to acquire flow data
Data analysis	Data was analyzed using FlowJo (v10), GraphPad Prism (v9), OMIQ (website: OMIQ.ai) and ClustVis (https://biit.cs.ut.ee/clustvis/)

For manuscripts utilizing custom algorithms or software that are central to the research but not yet described in published literature, software must be made available to editors and reviewers. We strongly encourage code deposition in a community repository (e.g. GitHub). See the Nature Portfolio guidelines for submitting code & software for further information.

Data

Policy information about availability of data

All manuscripts must include a data availability statement. This statement should provide the following information, where applicable:

- Accession codes, unique identifiers, or web links for publicly available datasets
- A description of any restrictions on data availability
- For clinical datasets or third party data, please ensure that the statement adheres to our policy

The datasets generated during and/or analysed during the current study are available from the corresponding author on reasonable request.

Human research participants

Policy information about studies involving human research participants and Sex and Gender in Research.

Reporting on sex and gender	n/a
Population characteristics	n/a
Recruitment	n/a
Ethics oversight	n/a

Note that full information on the approval of the study protocol must also be provided in the manuscript.

Field-specific reporting

Please select the one below that is the best fit for your research. If you are not sure, read the appropriate sections before making your selection.

[x] Life sciences   [ ] Behavioural & social sciences   [ ] Ecological, evolutionary & environmental sciences

For a reference copy of the document with all sections, see nature.com/documents/nr-reporting-summary-flat.pdf

Life sciences study design

All studies must disclose on these points even when the disclosure is negative.

Sample size	Mice were assigned randomly to groups and sample size is indicated in the figure legends. In general n=4-5 mice per group which is common for experiments with inbred mice and sufficient to determine statistical significance in previous helminth infection studies.
Data exclusions	No data were excluded
Replication	Reproducibility was confirmed by repeat experiments. All repeated experiments yielded similar results. The number of experiments performed can be found in the figure legends.
Randomization	Mice were randomly assigned to experimental groups
Blinding	No blinding was done as all mice were of the same strain. Infected animals required health monitoring and separate housing from control mice.

Reporting for specific materials, systems and methods

We require information from authors about some types of materials, experimental systems and methods used in many studies. Here, indicate whether each material, system or method listed is relevant to your study. If you are not sure if a list item applies to your research, read the appropriate section before selecting a response.

Materials & experimental systems

n/a	Involved in the study	
[ ]	[x]	Antibodies
[ ]	[x]	Eukaryotic cell lines
[x]	[ ]	Palaeontology and archaeology
[ ]	[x]	Animals and other organisms
[x]	[ ]	Clinical data
[x]	[ ]	Dual use research of concern

Methods

n/a	Involved in the study	
[x]	[ ]	ChIP-seq
[ ]	[x]	Flow cytometry
[x]	[ ]	MRI-based neuroimaging

Antibodies

Antibodies used	Described in Supplementary Table 1
Validation	Antibodies were validated by the manufacturers and used in accordance with the manufacturers recommendations.<br>Abcam: https://www.abcam.com/en-nl/technical-resources/guides/antibody-basics/how-to-choose-and-use-antibodies#antibodyvalidationinspecificapplicationsandspecies<br>BioLegend: "Each lot of this antibody is quality control tested by immunofluorescent staining with flow cytometric analysis."

BD Biosciences: "The production process underwent stringent testing and validation to assure that it generates a high-quality conjugate with consistent performance and specific binding activity."

Eukaryotic cell lines

Policy information about cell lines and Sex and Gender in Research

Cell line source(s)	L929 mouse-derived fibroblast cell line for the generation of supernatant containing macrophage colony stimulating factor (M-CSF)
Authentication	cell line was not authenticated
Mycoplasma contamination	mycoplasma testing was not peformed
Commonly misidentified lines (See ICLAC register)	no commonly misidentified cell lines were used

Animals and other research organisms

Policy information about studies involving animals; ARRIVE guidelines recommended for reporting animal research, and Sex and Gender in Research

Laboratory animals	C57BL/6JOlaHsd Mice were used aged 8-16 weeks. Mice were housed in a temperature-controlled room with a 12-hour light-dark cycle and ad libitum access to food and water under specific pathogen–free conditions.
Wild animals	No wild animals were used in this study.
Reporting on sex	Female and male mice were used but sex was not considered in the study design. Similar results were obtained with box sexes.
Field-collected samples	No field samples were collected for this study.
Ethics oversight	Experiments were complete in compliance with the Guide for the Care and Use of Laboratory Animal Research, and with approval from the Dutch Central Authority for Scientific Procedures on Animals (CCD; license number: AVD1160020198846)

Note that full information on the approval of the study protocol must also be provided in the manuscript.

Flow Cytometry

Plots

Confirm that:

- [x] The axis labels state the marker and fluorochrome used (e.g. CD4-FITC).
- [x] The axis scales are clearly visible. Include numbers along axes only for bottom left plot of group (a 'group' is an analysis of identical markers).
- [x] All plots are contour plots with outliers or pseudocolor plots.
- [x] A numerical value for number of cells or percentage (with statistics) is provided.

Methodology

Sample preparation	Peritoneal exudate was obtained by injection of 5ml cold PBS containing 2% FBCS and 2mM EDTA (ThermoFisher, 15575020) into the exposed abdomen of sacrificed mice, which was subsequently withdrawn after ~20 seconds of gentle agitation, and kept on ice until counting and plating.<br>Published protocols were adapted for isolation of leukocytes from the small-intestine55 and colon (including caecum)56. Both tissues were placed on PBS-soaked paper towel, opened longitudinally, and intestinal contents were scraped off using a metal spatula. Opened intestines were washed vigorously by shaking in a 50ml tube containing 15ml of Ca/Mg-free HBSS (ThermoFisher, 14170112) containing 2mM EDTA (wash media), then cut into 1-2cm pieces and stored in 10ml of wash media on ice until further processing. To remove mucous and strip epithelial cells, tissues were shaken vigorously in 50ml tubes with 10ml pre-warmed wash media, strained through 250µM Nitex, placed back in tubes containing 10ml fresh wash media, and incubated for 20 min at 37oC, shaking at 200rpm. Small intestines were washed a total of 3 times, colons a total of 2 times. Small intestine digest: 10ml RPMI containing 10% FBCS, 1mg/ml collagenase VIII (Sigma, C2139), 40U/ml DNAse I (Sigma, D4263) for 15-20min (or until tissue is not quite completely digested). Colon digest: 10ml RPMI containing 10%FBCS, 1mg/ml collagenase IV (Sigma, C5138), 0.5mg/ml collagenase D (COLLD-RO), 1mg/ml dispase II (Sigma, D4693), 40U/ml DNAse I for 25-30min. Digestion was stopped by immediate addition of cold RPMI containing 10% FBCS. Digested intestines were filtered through a 100µm strainer, spun at 400g for 5min, resuspended in FACS buffer (2% FBCS, 2mM EDTA) and filtered a second time through a 40µM strainer, spun, resuspended and stored on ice for counting and plating.<br>Liver digestion was also adapted from previously published protocols57. Livers were taken and placed in cold RPMI, minced with scissors and immediately transferred to tubes containing 10ml digest buffer (same as colon). Minced livers were digested for 25-30min at 200rpm, 37deg, with additional manual shaking vigorously by hand every 5-8 min. Digested livers were filtered through 100um strainers, and topped-up with cold RPMI containing 10% FCS up to 50ml, spun at 300g for 5min. Supernatant was removed, and cells were washed a second time with 30ml FACS buffer. The remaining pellet was treated

with ACK red-blood cell lysis buffer, washed and CD45 enriched using a MACS positive selection kit (Miltenyi, 130-052-301).
Lungs and spleens were placed in a 2ml tube with cold PBS, removed and minced in a new 2ml tube, before direct addition of 1.5ml digest buffer consisting of 1mg/ml collagenase IV and 40U/ml DNAse I and shaken for 30min before storing on ice, mashing through 100µM filter and washing with 10ml cold RPMI with 10% FBCS. Homogenates were red-blood cell lysed, washed, and passed through a 40µm strainer before counting and plating.
As described previously58, brain were digested after mincing with a razor in 0.2mg/ml Collagenase IV and 40U/ml DNAse I in a shaking waterbath for 1 hour. A 1ml pipet was used to further dissociate digesting brains every 15min. Homogenate was filtered through a 100uM strainer immediately before CD45+ MACS isolation.

A complete list of antibodies and dilutions is shown in Supplementary Table 1. Intracellularly stained metabolic targets were conjugated in-house using the corresponding kit according to the manufacturer's protocol. For in vitro experiments, all cells from a single well were stained after transferring to 96-well V-bottom plate. For tissues, 1-2x106 cells (numbers kept consistent within experiments) were stained in V-bottom plates in 50µl for each step. Cells were pre-stained with viability dye and Fc-block in PBS for 15min on ice. If required, subsequent live surface-staining was performed for certain targets in FACS buffer for 30 min on ice (Supplementary Table 1) before fixation. Cells were fixed with eBioscience Foxp3 fixation/permeabilization staining kit according the recommended protocol. Following fixation, remaining surface targets were stained in FACS buffer for 30min at 4oC. All staining was done in the appropriate buffer containing 1x Brilliant Stain Buffer Plus (BD Biosciences, 566385) and TrueStain Monocyte Block (BioLegend, 426103) . Cells were washed twice in perm/wash before staining intracellular targets in 1x permeabilization buffer, containing Fc-block, for 2 hour at 4 deg. If staining for RELMα, primary and secondary stains were completed (30min each) before metabolic target staining to prevent Fc-binding of the rabbit isotypes by the secondary.

Instrument	Cytek Aurora 5-Laser
Software	SpectroFlo version 3 (Cytek) was used for acquiring and unmixing, FlowJo (BD Bioscience) v10, OMIQ.ai and GraphPad Prism version 9.3 were used for data analysis and presentation.
Cell population abundance	Macrophage abundance (approx. mean freq. of CD45+) varied by tissue. PEC = 40%, Liver = 15%, Lung = 7%, Colon = 20%, Small intestine = 10%, Brain = 85%, Spleen = 1%.
Gating strategy	FSC-A vs SSC-A were used to exclude debris, followed by viability dye negative cells, single cells (FSC-H vs A) and CD45+ cells. Further gating varied depending on tissue and is shown in the supplementary data.

☒ Tick this box to confirm that a figure exemplifying the gating strategy is provided in the Supplementary Information.
